# Use of cornstarch or fermented soybean meal in lactation diet improved sows’ nutrient utilization and litter performance during lactation

**DOI:** 10.5713/ab.25.0106

**Published:** 2025-06-10

**Authors:** Li Zhe, Hongmei Wen, Fangyuan Chen, Yong Zhuo, Yan Lin, Shengyu Xu, Xuemei Jiang, Lingjie Huang, Lianqiang Che, Bin Feng, De Wu, Takele Feyera, Zhengfeng Fang

**Affiliations:** 1 Key Laboratory for Animal Disease-Resistance Nutrition of China Ministry of Education, Animal Nutrition Institute, Sichuan Agricultural University, Chengdu, China; 2 Provincial Key Agricultural Enterprise Research Institute of King Techina, Hangzhou King Techina Feed Co., Ltd., Hangzhou, China; 3 Department of Animal Science and Veterinary Sciences, Aarhus University, AU-Viborg, Tjele, Denmark

**Keywords:** Cornstarch, Fermented Soybean Meal, Growth Performance, Lactating Sows, Piglets

## Abstract

**Objective:**

This study investigated the effects of cornstarch (CS) and fermented soybean meal (FSM) substitutions for conventional corn and soybean meal, respectively, on sow’s performance, nutrient digestibility, milk composition, and oxidative status during lactation.

**Methods:**

Twenty-four lactating sows (8 sows/treatment) were assigned to either a standard lactation diet (CON), a CS diet in which pure CS substituted 60% of starch provided by conventional corn, or FSM diet in which FSM substituted 60% of crude protein provided by soybean meal. The experiment lasted from day 2 to 28 of lactation.

**Results:**

Compared with CON, the CS and FSM groups increased piglet live weight (p<0.05) and average daily gain (p<0.10), elevated milk superoxide dismutase and glutathione peroxidase (p<0.05), respectively, on day 28, and reduced (p<0.05) milk somatic cell counts on day 28; the CS group had higher (p<0.05) coefficient of apparent total tract digestibility of dry matter, gross energy, and ash but lower (p<0.05) serum β-hydroxybutyric acid and non-esterified fatty acids on day 28.

**Conclusion:**

In conclusion, the use of CS is much more robust in relieving body mobilization although both CS and FSM diets had beneficial effects on piglet performance by improving nutrient digestibility and milk quality of lactating sows.

## INTRODUCTION

The lactation period is a critical reproductive cycle in sow, influencing piglet growth and posing metabolic challenges to lactating sows. During this phase, a substantial amount of energy and nutrients is required for milk production [1–3]. Inadequate dietary energy and protein during lactation can lead to excessive body reserve mobilization, potentially compromising piglet growth and subsequent reproductive performance of sows [4–6]. Conversely, excessive fat mobilization may release free radicals into the systemic circulation [7], compromising sow antioxidant capacity and overall health [6].

In the optimization of standard lactation diet in the USA and many Asian countries, corn and soybean meal are the primary feedstuffs. Advances in feed processing technology have led to the use of cornstarch (CS) and fermented soybean meal (FSM) to enhance the utilization of energy in corn and protein in soybean meal. The apparent total tract digestibility of CS is as high as 100% when the particle size of corn powder is small enough [8]. Processing soybean meal into FSM can ameliorate the negative effects of trypsin inhibitors, reduce peptide size, increase protein availability, improve appetite, and enhance sow antioxidant capacity [9–11]. CS and FSM are two typical nutrient sources to regulate the sow lactation performance by promoting energy and protein efficiency, respectively. Consequently, incorporating CS and FSM into the diet for lactating sows could support lactating sows by optimizing nutrient requirements, reducing body reserve mobilization, and improving progeny growth. To date, limited studies have evaluated CS as the main energy feedstuff for lactating sows, nor has there been a comparison between energy- and protein-efficiency-promoting strategies involving the addition of CS and FSM, respectively.

We hypothesized that partially replacing corn with CS and soybean meal with FSM in the lactating sow’s diet can improve nutrient utilization and sow health while enhancing piglet growth during lactation. Accordingly, this study investigated the impact of substituting CS for conventional corn and FSM for standard soybean meal in the diet of lactating sows on nutrient digestibility, oxidative status, sow milk production, and piglet growth performance during lactation.

## MATERIALS AND METHODS

### Animals, diets, and feeding

A total of 24 multiparous sows (Landrace×Yorkshire, parity 4 and 8 sows/treatment) were included in the experiment from day 2 of lactation until weaning on day 28 of lactation. The sows were blocked for backfat thickness and randomly assigned to one of three dietary treatments as described below. The sows and their offsprings were housed in the farrowing crate until weaning. Each pen had a covered creep area with a heating lamp to provide the optimal climate for the litter. Litters were standardized to have 11 piglets each by cross-fostering within 48 h after farrowing and creep feeding was not provided for the piglets during the experimental period. The room temperature and the relative humidity were automatically controlled at 26°C and 60% to 70%, respectively.

From day 2 to 28 of lactation, sows were fed a corn-, wheat bran, and soybean meal-based standard lactation diet (CON) formulated according to the recommendation of National Research Council [12], with little modification for the other two treatment groups (Table 1). Sows in the CS treatment group were fed a diet similar to CON, but 200 g/kg pure CS was added to substitute 60% of starch provided by the basal corn in the CON diet. Moreover, extra soybean meal than in the CON diet was added to the CS group to keep similar crude protein content as in the CON diet. Sows in the FSM treatment group were fed a diet like the CON group, but 80 g/kg FSM was added to substitute 60% of crude protein provided by the basal soybean meal in the CON diet, thus the level of soybean meal was down-regulated to match the crude protein content with the CON diet. All three diets were formulated to be isoproteinic and isoenergetic.

On the first day of the experiment, feed was provided at 3 kg/d, and then increased gradually by 0.5 kg/d from day 3 to 7 of lactation, then followed by ad libitum feeding afterwards until weaning. Feed was provided manually three times per day of equal size, and sows had free access to drinking water throughout the experiment. Daily feed left over was recorded to determine the daily feed intake of the sows during the experimental period. The feed samples were collected during the manufacturing process and stored at −20°C until further analysis.

### Recording and sampling

Initial body weight and backfat thickness of the sows were recorded on day 2 of lactation while the final was recorded on the morning of day 29 of lactation after an overnight fasting. The B-mode ultrasonography (RENCO Lean-Meater 89372; Renco, Golden Valley, MN, USA) was used to measure the backfat thickness, which was recorded at 65 mm from the midline at the last rib on both sides, in triplicate. The individual live weight of the piglets was measured on days 2, 7, 14, 21, and 28 of lactation to record their weekly weight gain, which was in combination with the litter size used to predict the milk yield of the sow according to Hansen et al [13]. The milk utilization efficiency of the piglet was calculated as the ratio of piglet growth gain to milk yield of the sows (kg/kg) as described previously, in which the dead piglets were accounted for to calculate the milk utilization efficiency [14].

Milk samples were collected from the sows on days 14 and 28 of lactation after injection of 2 mL of diluted oxytocin (1 mL oxytocin plus 1 mL normal saline) into the ear vein to induce milk let down. Milk sample (20 mL/sow) was collected from the 2nd to 5th teats after wiping the teats with ethyl alcohol. The collected milk samples were stored at −20°C until further analysis.

Blood samples (about 10 mL/sow) were collected from the ear vein of the sows on days 14 and 28 of lactation in the morning after overnight fasting for 12 h. The blood samples were allowed to clot by leaving them undisturbed at room temperature and centrifuged at 1,300×g for 15 min at 4°C and serum was harvested and stored at −20°C until further analysis.

Between days 18 and 21 of lactation, about 50 g of fecal grab samples were collected from each sow both in the morning and the afternoon, and the fecal samples were pooled per sow. To prevent volatilization of nitrogen and to avoid bacterial contamination, 10 mL of 10% hydrochloric acid and three drops of methylbenzene were added per 100 g of fecal weight. The fecal samples were stored at −20°C until analysis, when the coefficient of apparent total tract digestibility (CATTD) of nutrients was determined using acid-insoluble ash as an internal marker.

### Chemical analyses

The serum samples were sent to the Institute of Animal Nutrition, Sichuan Agricultural University to detect the concentrations of albumin, total protein, aspartate aminotransferase, alanine aminotransferase, total cholesterol, triglycerides, non-esterified fatty acids, β-hydroxybutyric acid, creatinine, and urea using a fully automatic biochemical analyzer (HITACH 3100; Hitachi, Tokyo, Japan). In addition, the activities of superoxide dismutase, catalase, and glutathione peroxidase and the concentrations of total antioxidant capacity and malondialdehyde were measured according to the manufacturer’s instructions using ELISA kits (Nanjing Jiancheng Bioengineering Institute, Nanjing, China).

The milk samples were sent to the New Hope Hongya Dairy Testing Center (Meishan, China) to analyze the concentrations of dry matter, fat, protein, lactose, and urea nitrogen in the milk by infrared spectroscopy using a Milkoscan FT2 instrument (Milkoscan 4000; Foss, Hillerød, Denmark). The somatic cell counts were measured by infrared spectroscopy using a FossMaticTM FC instrument (FossMaticTM FM 5000; Foss). Prior to the analysis of oxidative status in milk, the milk samples were centrifuged at 8,000×g for 15 min at 4°C to collect the milk supernatant. Afterward, the harvested milk supernatant was used to measure the contents of superoxide dismutase, catalase, glutathione peroxidase, total antioxidant capacity, and malondialdehyde in milk supernatant using ELISA kits according to the manufacturer’s instructions (Nanjing Jiancheng Bioengineering Institute).

The fecal samples were dried to constant weight at 65°C in the oven, followed by grinding. The feed and fecal samples were analyzed for dry matter, gross energy, crude protein, ether extract, and ash contents according to AOAC [15]. Acid-insoluble ash in feed and fecal samples was analyzed following the method described by Brestenský et al [16] and used as an internal marker to determine the CATTD as follows:


(1) $$\text{Digestibility coefficient}=1-\frac{\text{Indicator in feed }(\%)}{\text{Indicator in feces }(\%)}\times \frac{\text{Nutrient in feces }(\%)}{\text{Nutrient in feed }(\%)}$$

### Statistical analysis

All statistical analyses were performed using the Proc MIXED procedure of SAS (SAS 9.4; SAS Institute, Cary, NC, USA) with a randomized complete block design. The sows or litters were the experimental unit when appropriate in the statistical model. The repeated measures procedure was used for repeated measurements on sows and piglet performance and milk yield with the best variance and covariance structure based on the smallest AIC and BIC. The PROC GLIMMIX procedure with gamma transformation was used when the residual variance did not fit the normal distribution. Statistical differences among treatments were determined by adjusted Tukey’s multiple range test. The relationship among serum biochemical indicators was analyzed using the Pearson correlation coefficients using the CORR procedure of SAS. Results are presented as LSMEANS with pooled standard error within the dietary treatments. The statistical difference was declared at p*<* 0.05 and tendency at 0.05≤p*<*0.10.

## RESULTS

### Performance of the sows and their offspring

There was no evidence for the dietary treatment effects on sow body weight and back fat at day 2 of lactation as well as body weight and backfat losses, feed intake, and milk yield of the sows during the lactation period, though feed intake (p*<*0.05) and milk yield (p*<*0.05) were increased with the progress of lactation (Table 2). Piglets suckled sows fed CS and FSM supplemented diets had greater live weight at weaning (p*<*0.05) and tended to have greater average daily gain during lactation (p*<*0.10) compared with those suckled sows fed the CON diet. Piglets’ live weight, average daily gain, and gain-to-milk yield ratio increase (p*<*0.05), whereas litter size tended to decrease (p*<*0.10) with the progress of lactation.

### Coefficient of apparent total tract digestibility of energy and nutrients

**Figure 1:**
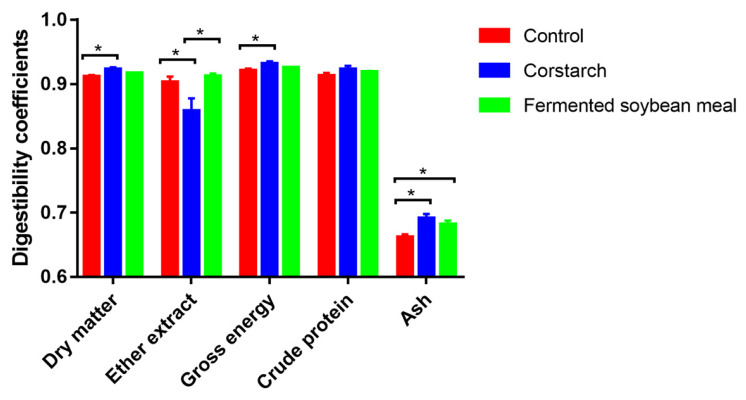
Impact of cornstarch and fermented soybean meal substitutions experimental on coefficient of apparent total tract digestibility of lactating sows. Acid-insoluble ash served as an internal marker for digestibility determination in this study. Values are illustrated with their SEM, while means labeled with * were differ within the same parameter (p<0.05). SEM, standard error of the mean.

The CATTD of dry matter, gross energy, and ash were greater (p*<*0.05; for all), whereas that of ether extract was lower (p*<* 0.05) in sows fed CS supplemented diet compared with sows fed the CON diet (Figure 1). Sows fed FSM supplemented diet had greater CATTD of ether extract than sows fed the CS supplemented diet (p*<*0.05).

### Milk composition

The study did not document any evidence for the dietary treatment effects on dry matter, fat, protein, lactose, and urea nitrogen compositions of sow’s milk (Table 3). However, milk somatic cell count on day 28 of lactation was greater in sows fed CON diet compared to milk from those fed CS and FSM supplemented diets (p*<*0.05), whereas sows fed CS supplemented diet tended to have greater milk somatic cell count on day 14 of lactation compared to those milk from sows fed CON and FSM supplemented diets (p*<*0.10).

### Serum biochemical indexes for sows’ body reserve mobilization and liver health

**Figure 2:**
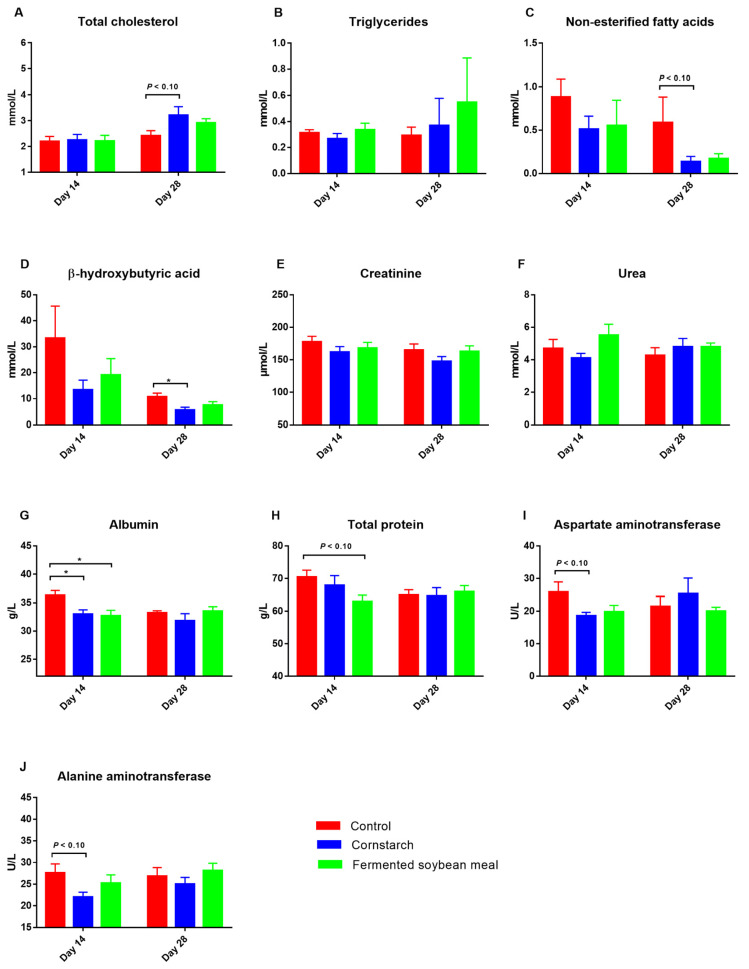
Impact of cornstarch and fermented soybean meal substitutions on serum biochemical indexes that are related with body reserve mobilization and liver health in lactating sows. Values are illustrated with their SEM, while means labeled with * were differ within the same day (p<0.05). SEM, standard error of the mean.

On day 14 of lactation, sows fed the CON diet had greater serum albumin concentration compared with those fed CS and FSM diets (p*<*0.05; Figure 2). Moreover, sows fed the CON diet tended to have greater serum total protein (p*<*0.10) compared to those fed the CS and FSM diets, and aspartate aminotransferase and alanine aminotransferase activities (p*<*0.10) compared with sows fed the CS diet on day 14 of lactation. Sows fed the CON diet had greater serum β-hydroxybutyric acid (p*<*0.05) but tended to have greater non-esterified fatty acids (p*<*0.10) and lower total cholesterol (p*<*0.10) concentrations on day 28 of lactation compared with sows fed the CS diet (Figure 2).

### Serum and milk antioxidant capacity

Serum activity of superoxide dismutase was lower in sows fed the CS diet both on day 14 (p*<*0.05) and day 28 (p*<*0.05) of lactation compared to the other groups (Table 4). Serum activities of glutathione peroxidase (p*<*0.05) and total antioxidant capacity (p*<*0.05) were lower on day 14 of lactation in sows fed the CS diet compared to sows fed the CON diet. Serum activity of glutathione peroxidase was greater in sows fed FSM diet on day 28 of lactation compared to those fed the CON diet (p*<*0.05). On day 28 of lactation, the activities of superoxide dismutase (p*<*0.05) and glutathione peroxidase (p*<*0.05) in milk supernatant were greater in sows fed the CS and FSM supplemented diets, respectively, compared to the other groups.

### Pearson correlation matrix among serum biochemical and antioxidant indexes

**Figure 3:**
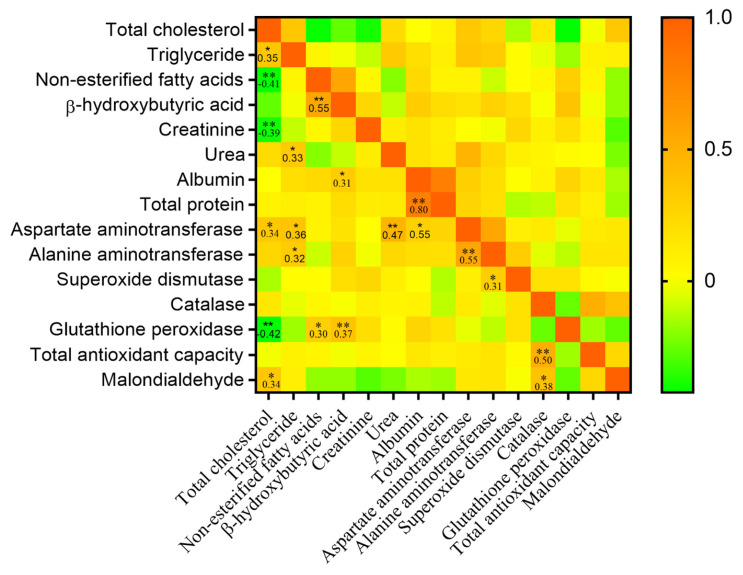
Pearson correlation matrix of serum biochemical and serum antioxidant indexes in lactating sows fed a diet cornstarch and fermented soybean meal substitutions. The significant correlations at p<0.05 and p<0.001 were marked with * and **, respectively, in the cells. The scores are colored according to the scale listed on the right.

The serum concentration of total cholesterol revealed negative correlations (Figure 3) with non-esterified fatty acids (r = −0.41; p*<*0.05), creatinine (r = −0.39; p*<*0.05), and glutathione peroxidase (r = −0.42; p*<*0.05). Conversely, a positive correlation emerged between the serum concentration of total cholesterol and triglycerides (r = 0.35; p*<*0.05), aspartate aminotransferase (r = 0.34; p*<*0.05), and malondialdehyde (r = 0.34; p*<*0.05). Moreover, β-hydroxybutyric acid demonstrated a strong correlation with non-esterified fatty acids (r = 0.55; p*<*0.05) and glutathione peroxidase (r = 0.37; p*<*0.05). Further, strong positive correlations were observed between urea and aspartate aminotransferase (r = 0.47; p*<*0.05), albumin and total protein (r = 0.80; p*<*0.05), aspartate aminotransferase and alanine aminotransferase (r = 0.55; p*<*0.01), as well as catalase and total antioxidant capacity (r = 0.50; p*<*0.05).

## DISCUSSION

The present study investigated the impacts of substituting part of starch and crude protein provided by corn and soybean meal in standard lactation diet, respectively, by pure CS and FSM supplementation on the performance of both the sows and their progeny during lactation. The results demonstrated the beneficial impacts of the present dietary strategies on both the sows and their litters compared to those fed on the standard lactation diet. Litter size at weaning and the weaning weight of the piglet are the major factors determining sow productivity in commercial herds. The present dietary strategies demonstrated an improved piglets’ growth during lactation. Piglet growth is driven by milk intake and milk quality. High feed intake is crucial for lactating sows to maximize milk production and reduce excessive depletion of body reserves during lactation [3,6]. Even though not statistically significant, the daily feed intake of the sows was improved by 8% to 10% (410 to 500 g/d) in those fed the CS and FSM diets compared to sows fed the CON diet. In the current study, the increased daily feed intake of sows should be the primary driving factor for milk yield improvement, thereby increasing piglet weaning weight through enhanced nutrient supply. Notably, the improved feed intake in the CS-fed sows not only contributed to higher milk production but also resulted in a numerically lower body weight loss (33%); this effect was absent in the FSM group. Consequently, serum β-hydroxybutyric acid and non-esterified fatty acids**—**catabolic products derived from body fat mobilization**—**were significantly lower only in sows fed the CS diet. The observed disparity in body condition between CS and FSM sows could originate from differential utilization of energy substrates: CS likely enhances carbohydrate-driven metabolic pathways, whereas FSM may prioritize protein-related catabolic processes. Carbohydrate is generally broken down via glycolysis and then enters Krebs’ cycle or oxidative phosphorylation to produce energy, whereas protein mainly contributes to providing functional amino acids rather than energy [17]. When compared with starch, the efficiency of protein as an energy source will be relatively lower since the energy contained in urea, the final metabolite of protein in swine, is excreted via urine. Furthermore, the greater digestibility of dry matter and gross energy observed in sows fed the CS diet supports its higher energy efficiency, which may partially explain the reduced body weight loss and lower circulating β-hydroxybutyric acid and non-esterified fatty acid levels. These findings highlight that divergent metabolic pathways in sows directly modulate both maternal metabolic efficiency and offspring performance.

Production of high quantity and quality of milk is one of the indicators of better lactation performance [18], with a subsequent positive impact on the growth of the piglets during lactation. Although milk yield was not statistically different, sows fed the CON diet had 5- and 4-fold greater somatic cell counts in milk on day 28 of lactation compared to milk from sows fed the CS and FSM diets, respectively. Increased somatic cell counts in milk could possibly imply the risk of bacterial infection in the udder, which could progressively develop into mastitis under the worst scenarios [19]. Thus, our result implied that udder health status was improved in sows fed the CS and FSM diets in the present study. An inverse relationship between antioxidant activities in milk and milk somatic cell counts was reported by Yigit et al [20]. In the present study, sows fed the CS and FSM diets had greater superoxide dismutase and glutathione peroxidase activities in milk supernatant, respectively, on day 28 of lactation, indicating their contribution to down-regulating the milk somatic cell counts. As reported by a previous study, antioxidants can be transferred from sows to their progeny via the milk, with this process mediated through the ingestion of maternal milk by offspring [21]. Taken together, the improved milk quality, characterized by higher antioxidant activities and lower somatic cell counts, should be another factor contributing to the increased piglet body weight in both the CS and FSM groups.

The improved digestibility of dry matter and gross energy observed in sows fed the CS diet in this study is consistent with the finding of Cervantes-Pahm et al [8]. However, the substitution of CS for starch in corn seemed to decrease the CATTD of ether extract, which may be related to the balance between C and N release rate and the mechanism behind this could not be explained at this stage. Furthermore, the increased digestibility of ash detected in sows fed the FSM diet is consistent with the findings of Wang et al [22] and Wang et al [10] that reported increased digestibility of Ca and P in sows fed a FSM-based diet. In contrast to our findings, Wang et al [10] observed increased energy and crude protein utilization in FSM-fed sows. This discrepancy could be attributed to the difference in the sources of FSM products and the feeding duration in these two studies.

Serum biochemical profiles are the potential biomarkers for the physiological and metabolic status in sows and these biochemical profiles are expected to be more dynamic during lactation due to high body reserve mobilization. As milk production is the highest priority in lactating sows, catabolic processes might reach at its peak during this period, consequently, increasing the concentration of ketone bodies formation in the circulation [1,23]. However, unlike in dairy cows, circulating ketone bodies do not bear as such metabolic disorder in sows [24]. Therefore, the lower serum concentrations of β-hydroxybutyric acid and non-esterified fatty acids on day 28 of lactation in sows fed the CS diet reflect a lower body reserve mobilization in this group, which is consistent with its lowest body weight loss during lactation period.

Serum concentrations of albumin and total protein are responsible for cellular osmotic pressure balance and substance transport [25] and closely associate with liver health [26]. In the current study, sows fed the CON diet showed greater serum concentration of albumin and tendency for greater serum total protein, aspartate aminotransferase, and alanine aminotransferase on day 14 of lactation compared to sows fed the CS and FSM diets. Moreover, the study further demonstrated a positive correlation of serum albumin with total protein and β-hydroxybutyric acid, suggesting that liver health was compromised in the CON group. In support to this, a previous study reported the negative implication of high blood aspartate aminotransferase and alanine aminotransferase concentrations on liver health in pigs [27]. Thus, sows fed the CS and FSM diets in the present study maintained better liver health status around the mid-lactation compared to sows fed the standard CON lactation diet.

The catabolic state during lactation is expected to challenge the systemic antioxidant capacity of the sows [6,7]. Mild to moderate oxidative stress is expected to stimulate the expression of antioxidant response-related genes and results in improved antioxidant states, whereas high or extreme oxidative stress will cause inflammation or apoptosis and lead to lower antioxidant activity [28]. Considering the better liver health status in sows fed the CS diet, the lower serum superoxide dismutase and glutathione peroxidase activities and total antioxidant capacity could be attributed to the lower oxidative stimulation. The positive correlation of serum glutathione peroxidase with non-esterified fatty acids and β-hydroxybutyric acid further substantiated the lower oxidative stimulation in sows fed the CS diet during lactation. Likewise, Zhao et al [29] observed linear decreases in blood superoxide dismutase and glutathione peroxidase activities in juvenile golden pompano that fed incremental CS (0%, 5%, 10%, 15%, 20%, and 25%). The above results indicate that sows fed the CS diet may have lower oxidative stress attributed to their lower body reserve mobilization.

## CONCLUSION

The present study demonstrated that supplementation of both CS and FSM in the diet of lactating sows improved maternal nutrient utilization and enhanced milk quality with the ultimate impact of better growth performance of the progeny during lactation. In addition, the use of CS increases energy utilization and decreases body reserve mobilization leading to improved liver health in sows during early stage of lactation. Taken together, the use of CS and FSM in the diet of lactating sows should be objective-oriented.

## Figures and Tables

**Table 1:** Dietary ingredients and calculated chemical composition of the experimental diets (as-fed basis)

Items	Treatments		

	Control	Cornstarch	Fermented soybean meal
Ingredients (g/kg)			
Corn	539.6	297.4	557.4
Wheat bran	83.2	82.7	83.0
Cornstarch^1)^	-	200.0	-
Extruded soybean	70.0	70.0	70.0
Fermented soybean meal^1)^	-	-	80.0
Soybean meal	156.0	200.0	61.8
Imported fish meal	20.0	20.0	20.0
Soybean oil	30.8	30.0	27.2
Soybean hull	50.0	50.0	50.0
L-Lysine.H_2_SO_4_ (70%)	0.34	-	0.56
Calcium hydrophosphate	11.8	12.4	10.9
Limestone	8.3	7.6	9.2
Vitamin and mineral premix^2)^	10.0	10.0	10.0
Functional premix^3)^	20.0	20.0	20.0
Nutritional level (g/kg)^4)^			
Dry matter	890.7	884.1	894.5
Gross energy (MJ/kg)	16.99	16.69	16.96
Digestible energy (MJ/kg)	15.64	15.55	15.69
Crude protein	160.3	164.6	160.7
Ether extract	72.0	43.4	72.5
SID amino acids			
Lysine	9.0	9.0	9.0
Methionine	2.5	2.3	2.5
Methionine+cysteine	5.2	4.9	5.1
Threonine	5.1	5.2	5.1
Tryptophan	1.7	1.8	1.6
Valine	6.8	6.8	6.8
Ash	55.6	53.8	54.4
Acid-insoluble ash	1.59	1.41	1.40
Calcium	8.0	8.0	8.0
STTD P	4.0	4.0	4.0

1) Cornstarch and fermented soybean meal (Jiangsu Yancheng Yuanyao Biological Technology) were brought from the market. The amino acid contents of FSM were (g/100 g): aspartate, 5.75; Threonine, 2.15; Serine, 2.23; Glutamate, 7.62; Proline, 3.26; Glycine, 2.14; Alanine, 2.23; Cysteine, 1.19; Valine, 2.27; Methionine, 0.71; Isoleucine, 2.32; Leucine, 3.82; Tyrosine, 2.13; Phenylalanine, 2.28; Lysine, 2.95; Histidine, 1.86; Arginine, 3.08; Tryptophan, 0.84.

2) Supplied per kilogram diet: vitamin A 7,000 IU; vitamin E 40 mg; vitamin D_3_ 4,000 IU; vitamin K_3_ 1.5 mg; vitamin B_2_ 4 mg; vitamin B_6_ 2.5 mg; niacin 25 mg; pantothenic acid 14 mg; iron (FeSO_4_·7 H_2_O) 150 mg; copper (CuSO_4_·5H_2_O) 25 mg; zinc (ZnO) 100 mg; manganese (MnSO_4_) 40 mg; iodine (Ca(IO_3_)_2_) 0.3 mg; selenium (Na_2_SeO_3_) 0.3 mg; Choline chloride 200 mg.

3) Supplied per ton of diet: DL-Methionine (98%) 300 g; L-Threonine (98%) 1,600 g; L-Valine mm (98%) 300 g; L-Tryptophan (98%) 100 g; L-Lysine sulfate (70%) 3,000 g; Antioxidant 150 g; 50% Choline chloride 1,000 g; Phytase 200 g; Organic chromium 200 g; Preservative 500 g; Baking soda 2,000 g; Sodium chloride 5,000 g.

4) The dry matter, gross energy, digestible energy, ether extract, ash, and acid-insoluble ash were analyzed values and others were calculated based on the expected value in the dietary ingredients.

SID, standardized ileal digestible; STTD P, standard total-tract digestible phosphorus; FSM, fermented soybean meal.

**Table 2:** Effect of cornstarch and fermented soybean meal substitutions on production performances of the sows and their offspring

Items	Treatments			SEM	Week in milk				SEM	p-value		
		
	Control	Cornstarch	Fermented soybean meal		1	2	3	4		Treatment	Week	Treatment× week
Sow												
No. of sows	8	8	8	-	-	-	-	-	-	-	-	-
Body weight on day 2 (kg)	215	215	212	7.9	-	-	-	-	-	0.914	-	-
Body weight loss on day 2–28 (kg)	21	14	20	4.4	-	-	-	-	-	0.507	-	-
Backfat on day 2 (mm)	13.5	13.5	13.4	2.34	-	-	-	-	-	0.977	-	-
Backfat loss on day 2–28 (mm)	1.84	1.76	1.69	0.682	-	-	-	-	-	0.984	-	-
Sow feed intake (kg/d)	4.99	5.49	5.40	0.244	2.70^d^	5.29^c^	6.10^b^	7.09^a^	0.185	0.115	<0.001	0.773
Sow daily milk yield (kg/d)	9.91	10.15	10.16	0.317	7.04^c^	10.54^b^	11.77^a^	10.95^ab^	0.234	0.812	<0.001	0.560
Litter size at weaning	10.6	10.4	10.4	0.17	10.8	10.6	10.4	10.3	0.16	0.614	0.075	0.992
Piglet average live weight (kg)	5.49^b^	5.96^a^	6.04^a^	0.199	2.98^d^	4.90^c^	6.90^b^	8.52^a^	0.214	0.019	<0.001	0.574
Piglet average daily gain (g/d)	227	259	261	11.4	186^b^	274^a^	287^a^	250^a^	10.4	0.067	<0.001	0.385
Piglet gain: milk yield (kg/kg)	0.26	0.27	0.27	0.005	0.29^a^	0.27^b^	0.25^c^	0.24^c^	0.005	0.207	<0.001	0.586

a–d Means within a row with different superscript differ (p<0.05).

**Table 3:** Effect of cornstarch and fermented soybean meal substitutions on milk composition of the sows

Items	Treatments			SEM	p-value

	Control	Cornstarch	Fermented soybean meal		
Dry matter (%)					
Day 14	21.13	21.75	19.31	0.059	0.336
Day 28	20.32	19.65	20.29	0.712	0.747
Fat (%)					
Day 14	8.41	9.20	6.97	0.133	0.325
Day 28	7.48	6.80	7.27	0.521	0.648
Protein (%)					
Day 14	5.17	5.03	4.65	4.958	0.366
Day 28	5.29	5.54	5.74	0.299	0.337
Lactose (%)					
Day 14	5.80	5.83	6.08	0.029	0.445
Day 28	5.69	5.78	5.74	0.143	0.875
Somatic cell count (×1,000 cells/mL)					
Day 14	344	621	312	101.4	0.091
Day 28	2,199^a^	453^b^	584^b^	496.6	0.014
Urea nitrogen (mg/dL)					
Day 14	52.4	55.1	49.5	3.29	0.476
Day 28	52.7	54.5	57.3	3.49	0.528

a,b Means within a row with different superscript differ (p<0.05).

**Table 4:** Effect of cornstarch and fermented soybean meal substitutions on antioxidant capacity of the sows during lactation

Items	Treatments			SEM	p-value

	Control	Cornstarch	Fermented soybean meal		
Serum					
Superoxide dismutase (U/mL)					
Day 14	31.47^a^	25.88^b^	30.87^a^	1.549	0.020
Day 28	28.99^a^	24.50^b^	31.04^a^	1.347	0.006
Catalase (U/mL)					
Day 14	5.17	4.28	4.41	1.272	0.831
Day 28	7.04	5.30	9.04	1.798	0.057
Glutathione peroxidase (U/mL)					
Day 14	2,322^a^	2,239^b^	2,255^ab^	21.8	0.032
Day 28	1,060^b^	1,259^ab^	1,463^a^	92.3	0.024
Total antioxidant capacity (mmol/L)					
Day 14	0.31^a^	0.27^b^	0.28^ab^	0.011	0.046
Day 28	0.30	0.29	0.31	0.011	0.468
Malondialdehyde (nmol/mL)					
Day 14	1.27	1.37	1.35	0.096	0.764
Day 28	1.37	1.48	1.52	0.079	0.438
Milk supernatant					
Superoxide dismutase (U/mL)					
Day 14	91.52	106.76	83.50	7.390	0.085
Day 28	50.15^b^	79.49^a^	55.06^b^	7.180	0.020
Catalase (U/mL)					
Day 14	2.83	2.54	2.15	0.764	0.768
Day 28	2.06	2.39	2.34	0.844	0.927
Glutathione peroxidase (U/mL)					
Day 14	48.40	54.50	39.32	6.052	0.188
Day 28	40.53^b^	42.95^b^	79.67^a^	8.853	0.007
Total antioxidant capacity (mmol/L)					
Day 14	1.05	0.84	0.71	0.195	0.360
Day 28	0.52	0.73	0.83	0.147	0.180
Malondialdehyde (nmol/mL)					
Day 14	2.97	3.42	2.45	0.362	0.161
Day 28	2.22	1.99	1.69	0.261	0.514

a,b Means within a row with different superscript differ (p<0.05).
